# A Clinical Case of Thymoma Presenting With Pure Erythroid Aplasia

**DOI:** 10.7759/cureus.76750

**Published:** 2025-01-01

**Authors:** Ana S Ramoa Oliveira, Joana Camões Neves, Ana Luís Vasconcelos, Filipa Rodrigues

**Affiliations:** 1 Internal Medicine, Hospital de Braga, Braga, PRT; 2 Gastroenterology, Hospital de Braga, Braga, PRT

**Keywords:** case report, mediastinal mass, paraneoplastic syndrome, pure red cell aplasia, thymoma

## Abstract

Thymic tumors are a rare condition that affects both genders equally, typically presenting between the ages of 40 and 70. Although often asymptomatic, they can give rise to paraneoplastic syndromes, with myasthenia gravis being the most common. The presentation of pure red cell aplasia, however, remains an uncommon manifestation. Thymectomy is the primary treatment, but it may not always be sufficient for managing associated syndromes, and immunosuppressive therapy may be necessary. This case involves a 50-year-old, otherwise healthy man, who presented to the emergency department with progressive dyspnea and asthenia over the course of one month. A mediastinal mass and hypoproliferative normocytic, normochromic anemia were identified, and after a diagnostic work-up, it was concluded to be a case of thymoma with associated pure red cell aplasia. The patient underwent surgery and radiotherapy and received immunosuppressive therapy. This case highlights a rare manifestation of an uncommon pathology, underscoring the need for a multidisciplinary approach and the challenges in treatment.

## Introduction

The thymus plays a crucial role in the development of adaptive immunity in humans [1,2]. Thymic epithelial tumors (TETs) comprise a heterogeneous group of rare mediastinal neoplasms, including thymomas, thymic carcinomas, and neuroendocrine tumors of the thymus [2,3]. The latest classification system for TETs follows the World Health Organization (WHO) classification of TETs, which was most recently updated in 2021 [2,3]. 

Thymoma is the most common primary neoplasm of the anterior mediastinum, affecting both men and women. The typical age of diagnosis is between 40 and 70 years, with no known risk factors. Thymomas exhibit indolent growth, and according to the World Health Organization, they can be morphologically classified into types A, AB, B1, B2, and B3 [2,3]. In addition to this morphological classification, prognosis is primarily determined by the Masaoka-Koga stage and the extent of resection (partial or complete) [3].

The majority of patients with thymoma are asymptomatic; however, chest symptoms may arise due to the tumor's growth and its involvement with adjacent organs [2]. Thymoma is also associated with various paraneoplastic syndromes, with myasthenia gravis being the most common, occurring in up to 50% of cases. Less commonly, pure red cell aplasia (PRCA), hypogammaglobulinemia, or Good's syndrome may manifest, with PRCA being described in approximately 5% of cases [2,4].

Paraneoplastic syndromes can manifest before, simultaneously with, or even after the diagnosis and treatment of thymoma. Although these associations are rare, limited literature is available, and the precise pathophysiological mechanisms underlying these syndromes remain incompletely understood [5].

The treatment of thymoma is individualized based on the stage of the disease and may involve surgical excision, chemotherapy, or radiotherapy, with the possibility of combining these modalities. Even with complete surgical resection, remission of the associated paraneoplastic syndrome is not always observed [3,5].

## Case presentation

A 50-year-old man presented to the emergency department with complaints of significant asthenia, dyspnea with minimal exertion, and palpitations, all of which had been present for one month. Two weeks earlier, he had sought medical care reporting similar symptoms. He was diagnosed with severe new-onset anemia, with a hemoglobin (Hb) level of 5.6 g/dL. He received a transfusion of three red blood cell (RBC) units and was discharged home. The patient denied chest pain, abdominal pain, fever, visible blood loss, skin changes such as jaundice, or any genitourinary or gastrointestinal issues.

On physical examination, cutaneous pallor was noted. The patient was hemodynamically stable, with a blood pressure of 120/66 mmHg and a heart rate of 84 beats per minute. There were no signs of heart failure, including no visible neck pulsations or elevation of the jugular venous pressure (JVP). There were no signs of respiratory distress, with an oxygen saturation of 95% on room air. Cardiopulmonary auscultation, as well as abdominal and rectal examinations, revealed no abnormalities. No neurological deficits were observed.

The laboratory results are presented in Table 1. They show prominent normocytic normochromic anemia. There were no abnormalities in the leukogram or platelet count. The reticulocyte production index was reduced. Iron studies were unremarkable, and levels of folic acid, vitamin B12, and haptoglobin were normal. Renal function was normal, with blood urea and creatinine levels within the reference range. Liver enzymes were also normal. Coagulation parameters were normal. Albumin levels were normal. C-reactive protein was within normal limits.

**Table 1:** Laboratory results and reference ranges.

Test	Result	Normal Range
Hemoglobin	7.8 g/dL	13.8-17.2 g/dL
Leukogram	6.500/µL	4,000-11,000/µL
Platelets	170.000/µL	150,000 -450,000/µL
Reticulocyte production index	1.2%	0.5-2.5%
Ferritin	100 ng/mL	20-500 ng/mL
Transferrin saturation	21%	20-50%
Folic acid	6.5 ng/mL	3.0-17.0 ng/mL
Vitamin B12	410 pg/mL	190-950 pg/mL
Haptoglobin	100 mg/dL	30-200 mg/dL
Blood Urea	5.5 mmol/L	2.5-7.1 mmol/L
Creatinine	1.0 mg/dL	0.6-1.2 mg/dL
Alanine aminotransferase (ALT)	36 IU/L	7-56 IU/L
Aspartate aminotransferase (AST)	40 IU/L	10-40 IU/L
International normalized ratio (INR)	1.0	0.8-1.2
Prothrombin time (PT)	12 seconds	11-14 seconds
Activated partial thromboplastin time (aPTT)	30 seconds	25-35 seconds
Albumin	4.2 g/dL	3.4-5.4 g/dL
C-reactive protein (CRP)	4.0 mg/L	<10.0 mg/L

**Figure 1:**
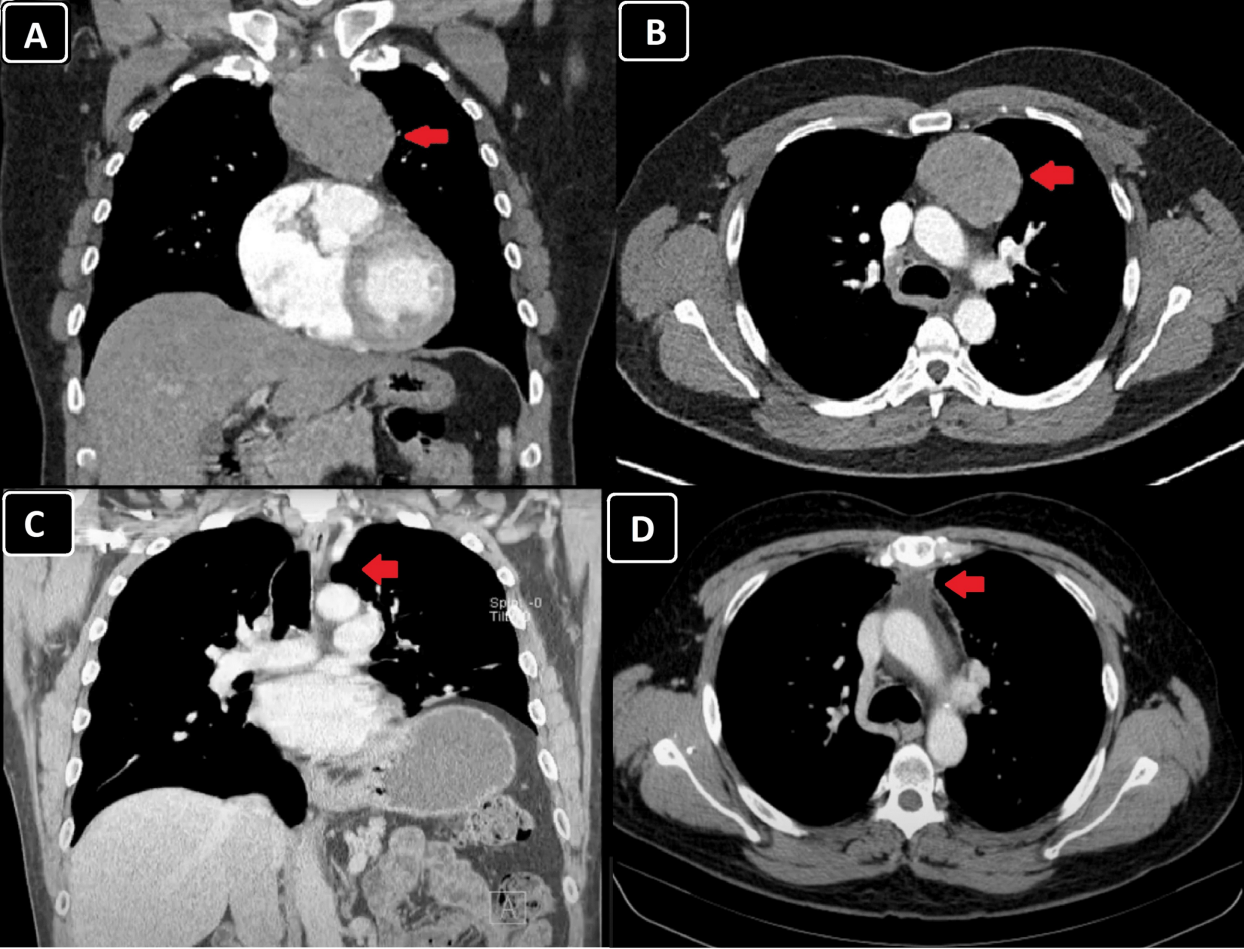
Computed tomography of the chest, in both (A) the coronal and (B) the axial planes, reveals an oval nodular lesion (red arrow) measuring approximately 7 cm in the longest axis and 4.5 cm in the shortest axis (on the axial plane), located in the anterior mediastinum. The lesion demonstrates heterogeneous density following the administration of contrast. The images C and D show the resolution of the thymoma.

A thoraco-abdomino-pelvic computed tomography (CT) scan revealed an oval nodular lesion measuring approximately 7 x 4.5 cm in the anterior mediastinum (Figure 1).

**Figure 2:**
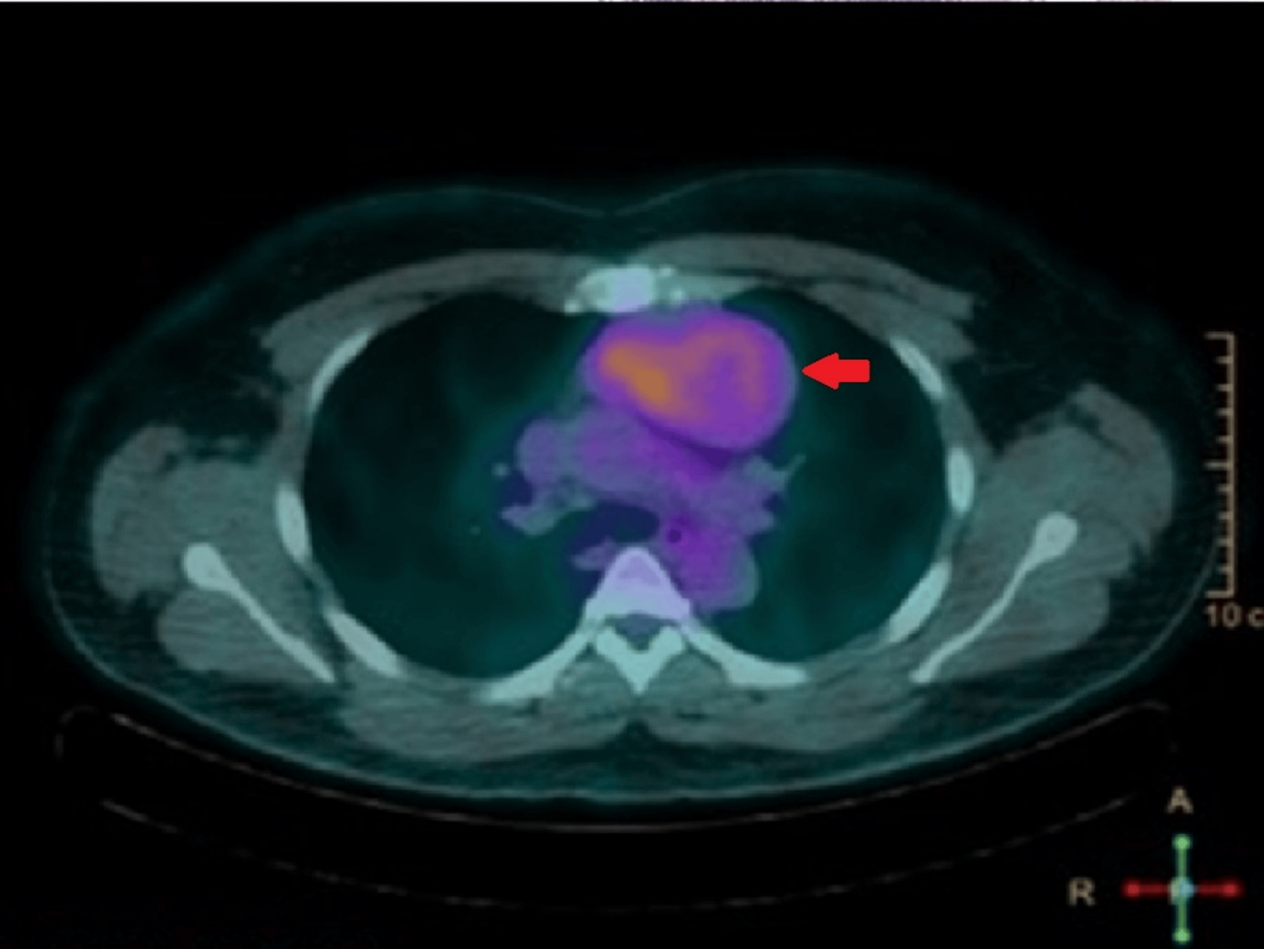
Positron emission tomography (PET): The lesion in the anterior mediastinum (red arrow) demonstrates heterogeneous increased uptake of 18F-FDG, with an SUVmax of 3.81.

The patient was admitted for the investigation of hypoproliferative normochromic normocytic anemia and a mediastinal mass. A biopsy of the mass was performed, and a comprehensive analytical study was completed. Key findings included a sedimentation rate of 46 mm/hr, with serum protein electrophoresis showing no abnormalities. Tests for human immunodeficiency virus (HIV), cytomegalovirus (CMV), Epstein-Barr virus (EBV), and parvovirus B19 were performed, including serological assays for HIV antibodies, CMV IgM and IgG, EBV VCA IgM and IgG, and parvovirus B19 IgM and IgG. All tests were negative. In addition, a positron emission tomography (PET) scan was performed, revealing a lesion in the anterior mediastinum with heterogeneous glycolytic metabolism and bilateral cervical lymph nodes exhibiting high metabolic activity, without other significant changes (Figure 2).

**Figure 3:**
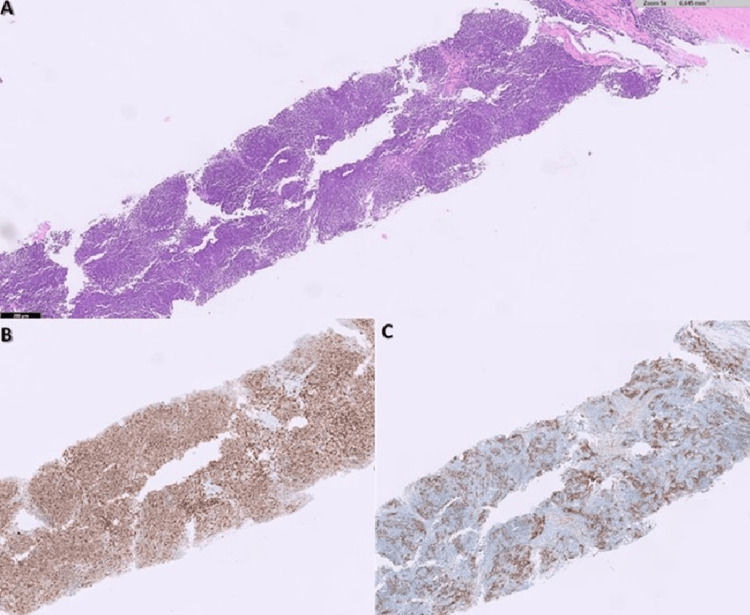
Thymoma biopsy. Image A (Hematoxylin and eosin (HE) stain, 50x magnification): HE staining reveals fibrous tissue infiltrated by a neoplastic process, characterized by small lymphocytes and a few epithelial cells. Image B (CD5 stain, 50x magnification): CD5 immunohistochemistry highlights the T lymphocytes within the neoplasm, confirming their involvement. Image C (CAM5.2 stain, 50x magnification): CAM5.2 immunohistochemistry demonstrates the epithelial cells within the neoplasm, indicating the biphasic nature of thymomas.

**Figure 4:**
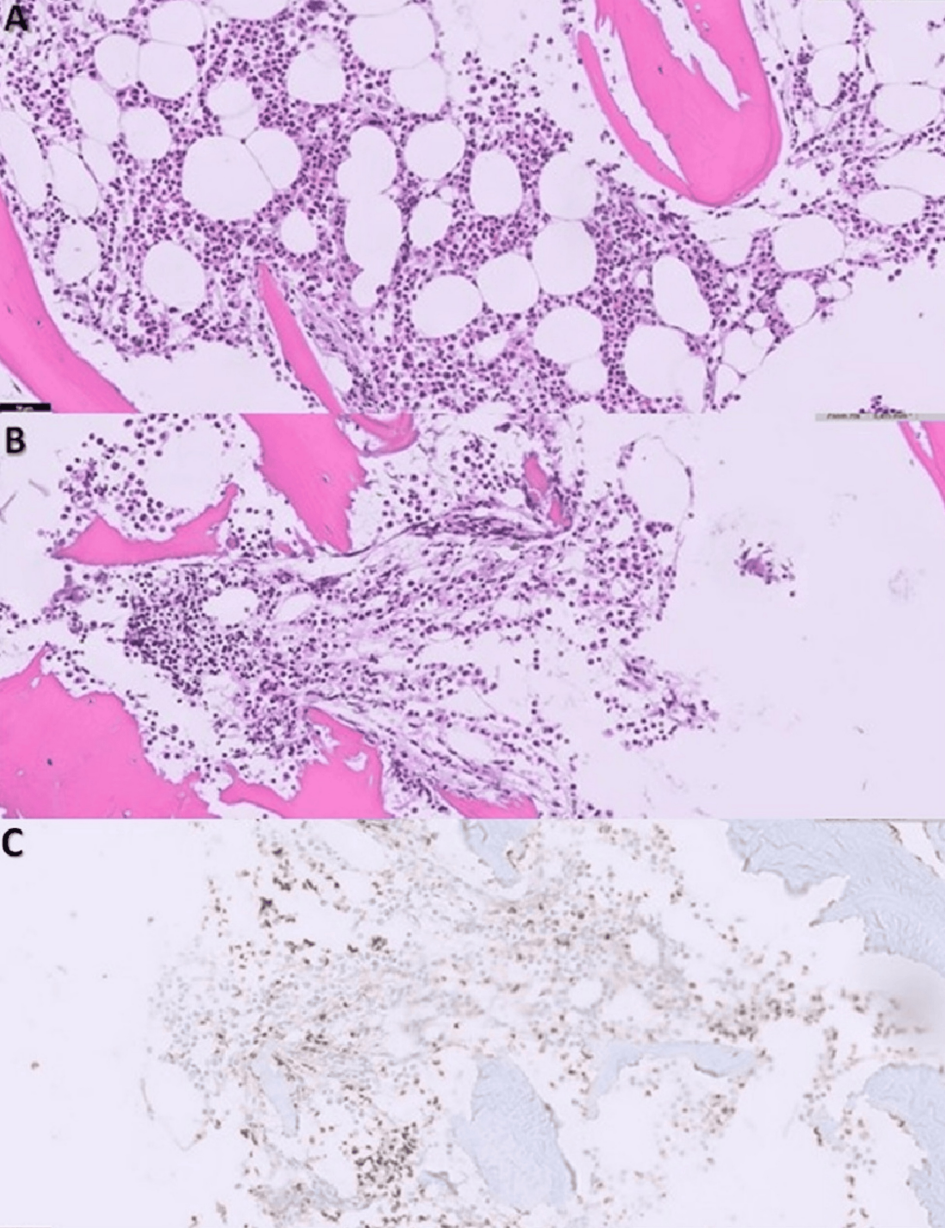
Bone marrow biopsy. Image A (Hematoxylin and eosin (HE) stain at 200x magnification): The intertrabecular space contains hematopoietic marrow with noticeable myeloid hyperplasia (increased myeloid precursor cells). There is a leftward deviation in myeloid maturation, meaning there is an overrepresentation of immature myeloid cells. Rare megakaryocytes (large cells responsible for platelet production) are visible. Image B (HE stain at 200x magnification): Hematopoietic marrow in the intertrabecular space is evident but shows rare erythroid lineage elements (reduced red blood cell precursors). This indicates a potential underrepresentation of erythropoiesis (red blood cell production). Image C (Glycophorin immunohistochemical staining at 200x magnification): Glycophorin immunohistochemical staining highlights erythroid lineage elements, enabling their clear identification within the marrow. This helps to confirm the presence and distribution of erythroid precursors.

The histological result of the mediastinal mass biopsy became available, confirming the diagnosis of thymoma (Figure 3). Tests for anti-acetylcholine receptor, anti-muscle-specific tyrosine kinase, and anti-LRP4 antibodies were negative. Immunoglobulin levels were normal (IgG 1200 mg/dL, IgA 250 mg/dL, IgM 200 mg/dL). A bone marrow aspiration was performed, showing no erythroid precursors in the smear. No blasts or extramedullary elements were observed, and no other significant findings were noted. The bone marrow biopsy revealed a paucity of hematopoietic marrow, with very few erythroid precursors and reactive changes in the myeloid lineage (Figure 4).

Throughout the hospitalization, the patient continued to require weekly RBC transfusions.

Following the diagnosis of pure red cell aplasia (PRCA) secondary to thymoma, the patient underwent an uneventful anterolateral thymectomy via median sternotomy. The anatomopathological study revealed a type AB thymoma, classified as Masaoka-Koga stage IIb due to invasion of the mediastinal pleura. The pTNM staging after thymectomy was determined as pT1b N2 R0, with a radial margin of less than 1 mm.

Ten days post-surgery, the patient required an additional RBC transfusion and was discharged for follow-up in Internal Medicine consultation and day hospital care for ongoing evaluations and weekly transfusions.

The case was presented and discussed in a multidisciplinary meeting involving Internal Medicine, Pneumology, Radiation Oncology, and Hematology. It was decided to proceed with adjuvant radiotherapy for approximately one month, comprising a total of 25 sessions. A follow-up chest CT scan performed four months after surgery showed no evidence of tumor recurrence (Figure 1).

Six months post-surgery, the patient did not show a resolution of PRCA. As a result, corticosteroid therapy was initiated with prednisolone at 1 mg/kg/day for 4 weeks. However, there was no response, and the need for weekly transfusions persisted. Consequently, cyclosporine was introduced at approximately 3 mg/kg/day, leading to a positive response. Two months after this combination therapy, there has been no need for additional transfusions, and the dose of prednisolone is being gradually reduced.

## Discussion

Thymomas are relatively rare tumors of the mediastinum, presenting with variable histological characteristics, diverse clinical manifestations, and differing patterns of progression. The thymus plays a vital role in the immune system, where the maturation and differentiation of hematopoietic precursor cells into T cells occurs. Although thymic function diminishes with age, evidence suggests that it is maintained in adulthood, and this maintenance may contribute to the association of thymus neoplasms with immunological and autoimmune disorders [1,5].

In the present case, the initial signal of the disease was anemia, attributed to PRCA. Among patients diagnosed with thymoma, less than 10% develop PRCA, and fewer than 5% of PRCA patients have a thymoma [6]. The pathophysiology of PRCA associated with thymoma remains incompletely understood, with several potential mechanisms proposed, including the presence of autoreactive T cells, a clonal disorder of lymphocytes, and the production of anti-erythroblast antibodies [7,8]. PRCA's anemia is typically normocytic and normochromic, characterized by erythropoietic insufficiency while granulopoiesis and megakaryopoiesis remain preserved [7,8].

According to the morphological classification system of the WHO, we present a case of type AB thymoma, which represents 25% of all thymomas [2,3]. This type exhibits a combination of spindle cells without atypia, epithelioid cells, and lymphocytes. However, this classification has not shown significant prognostic relevance. The Masaoka-Koga classification, based on postoperative staging, is preferred. In this case, the tumor is classified as stage IIb, due to macroscopic invasion of the mediastinal pleura [9].

The association between the histological type of thymoma and PRCA is not well-defined [10]. While Masaoka et al. reported that all 17 patients with thymoma and PRCA had spindle-shaped cells, subsequent studies have primarily linked PRCA with type B or AB thymomas [10].

Thymectomy is the cornerstone of treatment for thymoma and can be performed alone or as part of a multimodal approach, alongside radiotherapy or chemotherapy. In this case, complete tumor resection was conducted, improving the patient's prognosis. Postoperative radiotherapy was also administered. Although the evidence is limited, it is recommended in cases with invasion of adjacent structures or microscopic resection margins. Some studies have suggested a significant reduction in recurrence rates following radiotherapy [11].

Several paraneoplastic syndromes associated with thymoma may respond to thymectomy; however, the response to autoimmune PRCA has been inconsistent, with an initial remission rate of approximately 30% following surgery [12,13]. In addition to supportive therapy, adjuvant treatment with immunosuppressive agents may be required [12,14]. If erythropoiesis does not recover within the first 6 months after surgery, immunosuppressive therapy is recommended. The typical first-line treatment involves corticosteroid therapy, with prednisolone administered at 1 mg/kg/day for approximately 4 weeks. If the patient remains unresponsive, a combination of cyclosporine, cyclophosphamide, or another immunosuppressive agent may be considered. Some studies have shown favorable outcomes with cyclosporine, achieving remission in around 95% of patients [12-14]. In this case, thymectomy alone did not lead to the recovery of hematopoiesis within six months after surgery. This was also the experience of Tara Seibert et al. in their investigation, which does not support thymectomy alone as the primary treatment for PRCA [13]. As a result, prednisolone was initiated but proved ineffective. Subsequently, cyclosporine was introduced, resulting in positive outcomes, with regular patient follow-up. Generally, patients may receive cyclosporine for several months or until clinical remission is achieved.

It is recognized that the stage of the disease and the potential for complete regression are the most crucial prognostic factors. Consequently, Masaoka-Koga stage I and II thymomas are considered to have the best prognosis. However, the average survival for patients with associated PRCA is approximately 12 years, with most deaths attributed to the adverse effects of immunosuppression or PRCA refractory to therapy. According to the latest Spanish Society of Medical Oncology (SEOM), Spanish Group for Lung Cancer (GECP), Spanish Group for Thoracic and Hepatic Interventional Tumors (GETTHI) clinical guidelines for treating patients with TETs, regular radiological evaluations are essential. For stage I-II thymomas, CT scans are recommended three to four months after surgical resection, followed by annual CT scans for the first five years and subsequently every two years, with follow-up continuing for 10 to 15 years [15].

## Conclusions

The authors present a rare case of thymoma associated with PRCA, emphasizing the importance of considering a thymic neoplasm in patients presenting with normocytic, normochromic, hypoproliferative anemia. A multidisciplinary approach is crucial, as the treatment of paraneoplastic syndromes remains challenging. This case highlights the limited literature and lack of extensive scientific evidence due to the clinical rarity of the condition, which complicates the management approach. These patients require prolonged and regular follow-up, underscoring the need for continued surveillance for paraneoplastic syndromes that may develop even after thymectomy.
